# Incidence of Scrub Typhus according to Changes in Geographic and Demographic Characteristic in the Chungcheong Region of Korea

**DOI:** 10.3390/tropicalmed9070147

**Published:** 2024-06-30

**Authors:** Sungchan Yang, Gemma Park, Yuna Kim

**Affiliations:** 1 Division of Infectious Disease Response, Capital Regional Center for Disease Control and Prevention, Seoul 03741, Republic of Korea; npros33@korea.kr; 2 Division of Infectious Disease Response, Chungcheong Regional Center for Disease Control and Prevention, Seo-gu, Daejeon 35208, Republic of Korea; apile12@korea.kr

**Keywords:** scrub typhus, demographic characteristics, risk factor, tsutsugamushi

## Abstract

To ascertain the incidence trends of scrub typhus in the Chungcheong region, we analyzed the epidemiological survey information of 14,379 cases of scrub typhus reported to the integrated disease health management system of the Korea Centers for Disease Control and Prevention between 2012 and 2022, along with demographic data from the Korean Statistics Information Service. Geographical analyses were performed to confirm the correlation between high-risk areas and the proportion of elderly people. The average age, proportion of elderly people, and changes in the agricultural population were statistically associated with incidence. The incidence of scrub typhus, and the agricultural population, in the Chungcheong region has decreased compared with that in 2012–2013. However, recent trends indicate a resurgence linked to increased outdoor activity, with higher risks observed in older age groups. Additionally, advancing age correlates with a heightened probability of reinfection and additional infections with other febrile diseases. The incidence of scrub typhus in the Chungcheong region (2012–2022) is changing according to age and route of infection, highlighting the need for revised prevention and promotion policies.

## 1. Introduction

Scrub typhus is a typical febrile disease that is prevalent during the autumn season in Korea [1,2]. Approximately 1 billion people worldwide are exposed to risk of infection, and at least 1 million scrub typhus cases occur annually [3,4]. Scrub typhus is transmitted by the bite of chigger mite larvae infected with *Orientia tsutsugamushi*, often leaving a characteristic eschar at the part of the bite [5,6,7]. The primary symptoms include high fever or myalgia, headache, chills, gastrointestinal and lymphadenopathy symptoms. Infection can lead to several serious complications affecting the lungs, liver, brain, and kidneys, potentially resulting in multi-organ failure and death [8,9].

Scrub typhus is prevalent along the east coast of Russia in the north and northern Australia in the south [10]. However, recent studies have also noted its occurrence in Africa and Chile. Both residents and visitors in the high-risk area (India, Indonesia, and northern Australia), especially in rural regions, have a high probability of infection [11]. In 2016, more than 11,000 new cases were reported in Korea with over 4000 new cases documented annually since then. The disease predominantly affects individuals aged 60 years and older [12] with more than half of infection cases reported in the autumn season (weeks 44 to 47) [1]. In 2022, the Gyeongnam area reported the highest number of scrub typhus cases (1333 cases), which was followed by the Jeonnam area (1020 cases). However, the incidence rate per 100,000 people was highest in the Jeonnam area (554 cases), which was followed by the Jeonbuk area (416 cases), and it was higher in the south region compared with other regions of Korea [13]. In another study, the Chungcheong region had a high infection rate from collected mites [14]. 

Scrub typhus outbreaks are influenced by several factors. (1) Population of chigger mites: Most of the febrile cases infected during the autumn season are attributed to scrub typhus [15]. As such, a consistent correlation was observed between the relative increase in chigger mite populations and the trend of case reports. Previous studies have also reported a correlation between the number of chigger mites and scrub typhus cases [13,16]. (2) Environmental exposure: Cases of scrub typhus have recently emerged in urban areas due to activities like weekend farming, hiking, and clubbing [17,18]. Notably, the COVID-19 pandemic and the subsequent implementation of social distancing policy have influenced recreational preferences, prompting more visits to forests and engagement in activities such as camping and hiking in the habitats of chigger mites, contributing to higher bite rates [19]. (3) Climate factors: Suitable climatic conditions can create favorable environments for the reproduction and survival of rodents and chigger mites. One study reported that a 1 °C increase in the monthly average temperature, relative humidity (RH), and precipitation resulted in increases in the number of scrub typhus cases [20]. A previous study on risk factors of scrub typhus in Guangzhou showed similarly data that connected climate factors and scrub typhus cases [21,22]. (4) Vector: Rodents are the primary reservoirs for scrub typhus, and their density is closely associated with the number of scrub typhus cases [23]. The advent of artificial intelligence and machine learning has facilitated the analysis and prediction of various diseases, including scrub typhus. Analyzing its influencing factors is essential for its prevention [24]. In previous studies, various models (BRT, ARIMA, ANN) have been developed to forecast the climate factors of scrub typhus [25,26].

Further epidemiological studies are necessary to define the indisputable risk factors for scrub typhus, supporting public health interventions aimed at disease prevention and control [15].

Despite the clinical relevance of vector-borne diseases, comprehensive epidemiological studies and research investigations remain limited in South Korea. Therefore, we analyzed the factors affecting case occurrence through long-term case monitoring in a specific area. The Chungcheong region has a high incidence rate in Korea, and its demographic characteristics have significantly changed following the establishment of an administrative-centered complex city. We aimed to identify the key determinants of scrub typhus occurrence over the past 11 years using an epidemiological survey data with a focus on factors such as age, sex, agricultural population, and occupation. Additionally, we reevaluated the correlation between the incidence rate and demographic characteristics. 

This study also aimed to identify the factors contributing to the changes in the occurrence of scrub typhus based on the demographic characteristics according to region and to facilitate the effective management of outbreaks in the future. 

## 2. Methods

### 2.1. Data Collection

The Chungcheong region is located in the center of Korea in the shape of a butterfly and consists of the Daejeon, Sejong, Chungbuk, and Chungnam areas. Daejeon and Sejong are located in the center of the Chungcheong region, while Chungbuk is in the east and Chungnam is in the west area (Figure S1). The demographic data were obtained from the Korean Statistics Information Service and the Ministry of the Interior and Safety. Case and mortality data were derived from reports and epidemiological surveys of 14,379 patients with scrub typhus in the Chungcheong region (Daejeon, Sejong, Chungnam, and Chungbuk area) from 2012 to 2022. These were collected from the Integrated Disease Health Management System of the Korea Disease Control and Prevention Agency (KDCA).

### 2.2. Statistical Analysis

The association of demographic factors (sex, age, and occupation) with scrub typhus cases (incidence, death, reinfection, and additional infection of febrile diseases) was statistically analyzed using Pearson’s correlation analysis. Logistic regression was employed to predict the relative risk based on age, sex, region, and year. Statistical analyses were performed using IBM SPSS ver. 22.0 (IBM Corp., Armonk, NY, USA) and R 4.3.3.

The age categories were infants (0–12 years), teenagers (13–18), youth (19–39), seniors (40–59), elderly people (60–74), and those over 75, and were analyzed according to activity characteristics such as occupation.

Geographical analysis was performed using QGIS 3.34.2. The incidence of scrub typhus in cities by year was visually compared. Additionally, the association between high-risk areas for scrub typhus and regions with a high proportion of people aged 60 years or <18 years was determined.

### 2.3. Ethics Statement

The study was approved by the Institutional Review Board (IRB) of the KDCA (no. KDCA-2024-06-08). The requirement for informed consent was waived by the IRB.

## 3. Results

### 3.1. Demographic Characteristics by the Region

The population in Korea increased from 50.9 million in 2012 to 51.8 million in 2019, but since then, it decreased continuously to 51.4 million in 2022. In the Daejeon area, the population reached 1.52 million people in 2012 but declined after 2014, reaching 1.44 million in 2022. In the Chungnam area, the population has been declining since 2018 but increased to 2.12 million in 2022 compared with 2.02 million in 2012. In the Chungbuk area, the population increased from 1.56 million in 2012 to 1.59 million in 2019, but it has been declining since 2021. Unlike other regions, the Sejong area has seen a steady increase in its population, more than tripling from 110,000 in 2012 to 380,000 in 2022 (Figure S2).

**Figure 1:**
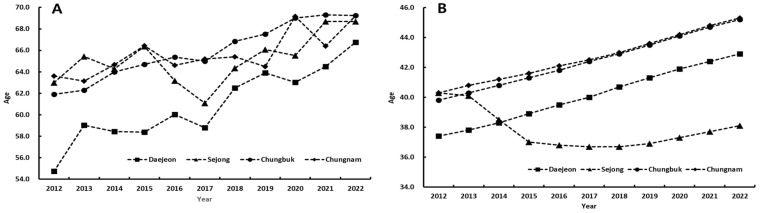
Changes in average age of patients with scrub typhus and average age by region. (**A**) Average age of case with scrub typhus. (**B**) Average age following the region.

The average age in the Chungcheong region from 2012 to 2022 increased from 37.4 to 42.9 years in Daejeon, from 39.8 to 45.2 years in Chungbuk, and from 40.3 to 45.3 years in Chungnam. Whereas, the average age in Sejong decreased from 40.3 years in 2012 to 36.7 years in 2017 and then slightly increased to 38.1 years in 2022 (Figure 1). 

**Figure 2:**
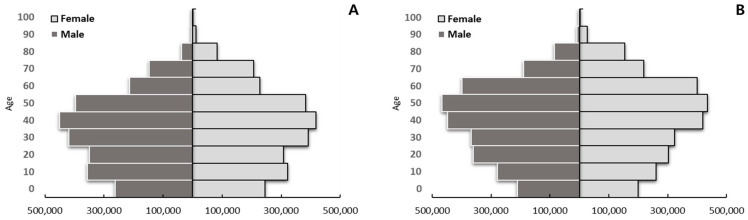
Age and sex proportion change in Chungcheong region between early years (2012–2015) and late years (2016–2022). (**A**) Proportion change in 2012–2015. (**B**) Proportion change in 2016–2022.

In terms of sex ratio in the Chungcheong region, the average male to female ratio was 1.2:1 between 2012 and 2016, but it increased to 2.3:1 times between 2018 and 2022. However, after the age of 60, there are consistently more females than males. In the age-specific analysis, the proportion in their 50s–60s tended to increase significantly in 2018–2022 and 2012–2016, while the proportion of under 40 decreased (Figure 2). 

### 3.2. Outbreak Status and Characteristics of Scrub Typhus in Chungcheong Region

**Figure 3:**
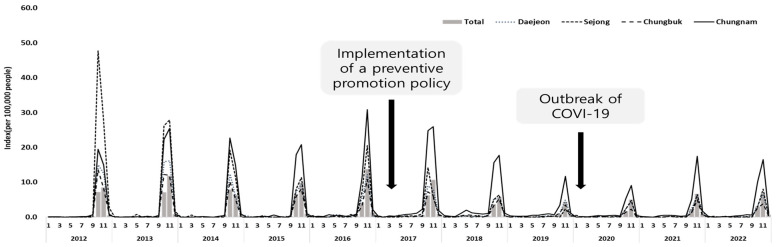
Monthly incidence of scrub typhus per 100,000 people by region.

From 2012 to 2022, 14,379 cases of scrub typhus were reported in the Chungcheong region with the highest number of cases in the Chungnam area (8617), which was followed by the Daejeon (2896), Chungbuk (2318), and Sejong areas (548). Similar to the national trends, the monthly incidence rate typically increased in September and peaked in November. The incidence rate decreased after 2017 due to the implementation of active prevention and promotion policies. However, the incidence has gradually increased since 2021 (Table 1). In the Sejong area, the incidence rate was 47.7 in November 2012, which was approximately six times higher than the national incidence rate (7.2). In 2013, the incidence rate was 27.8, which was more than twice as high as the national rate of 11.8. The Chungnam area had the highest incidence rate in the Chungcheong region since 2014, reaching 30.8 in November 2016, which is approximately twice as high as the national rate of 13.8 (Figure 3). 

When the incidence rate by sex was analyzed, females consistently had a higher rate than males. In 2012, the incidence rate was 69% higher in females than in males, but decreased slightly to a rate that was 35% higher than that of males in 2022.

By age group, those aged 60–74 years had the highest percentage of cases each year from 2012 to 2022 followed by those aged 40–59 years. However, those over 75 years demonstrated a higher rate than those aged 40–59 years after 2018. By occupational groups, the incidence rate of scrub typhus was higher among the farmer and fisher group, housewives (including farm families), and the unemployed with the proportion of the farmer and fisher group gradually decreasing (Table 1).

### 3.3. Characteristics of Deaths Associated with Scrub Typhus in the Chungcheong Region

From 2012 to 2022, 22 death cases due to scrub typhus were reported in the Chungcheong region with the highest number of cases occurring in 2022 (seven cases). The Chungnam area had the highest number of death cases (16), while Sejong reported no death cases during the survey period (Table 2). 

Regarding sex, the mortality rate was higher in males, with 13 deaths out of 5569 cases (0.2%) and nine deaths out of 8810 cases (0.1%) in females. Most of those who died were more than 75 years old except for three individuals: two in the 40–59 group and one in the 60–74 group. Sepsis is the main cause of death, excluding the direct effects of scrub typhus with factors such as multiple organ failure and respiratory failure showing a decreasing trend in recent years. The average time from symptom onset to death varied annually; however, most deaths occurred within 15 days of symptom onset (Table 2).

### 3.4. Correlation with Demographic Characteristics and Incidence of Case

The average age in most regions increased from 2012 to 2022, with the Daejeon area increasing by 5.5 years, the Chungbuk area by 5.4 years, and the Chungnam area by 5.0 years. However, in the Sejong area, the average age decreased by 2.2 years. The average ages of cases with scrub typhus also increased by 12.0 years in Daejeon, 7.3 years in Chungbuk, 5.6 years in Chungnam, and 5.7 years in the Sejong area during 2012–2022 (Figure 1). 

**Figure 4:**
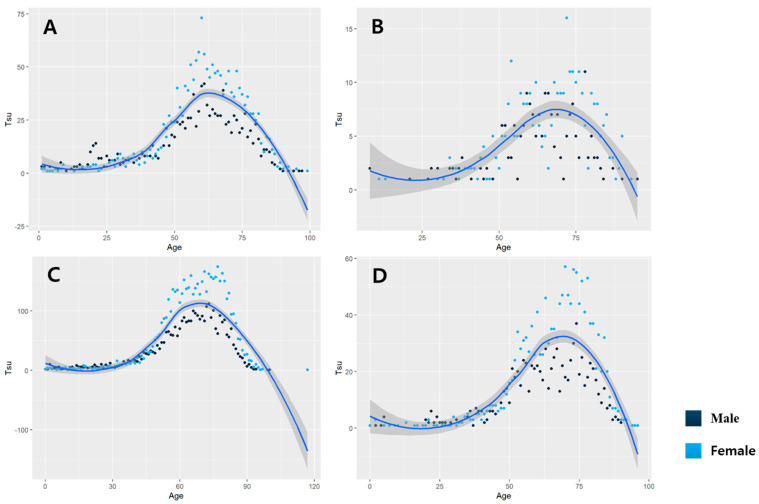
Analysis of the correlation between age and incidence by region. (**A**) Daejeon area. (**B**) Sejong area. (**C**) Chungnam area. (**D**) Chungbuk area.

The analysis of the correlation between age and case incidence by region showed that the incidence increased with age until approximately the 60s and then eventually decreased in the Daejeon area. Females exhibited a higher incidence than males between the ages of 50 and 75 years. In the Sejong area, the incidence tended to increase with age until approximately 70 years with a less pronounced difference between males and females; however, after the age of 75 years, females tended to have a slightly higher incidence. In the Chungnam area, the incidence increased with age until around 75 years, and females showed a higher incidence than males in the 60–90-year age group. In Chungbuk, the incidence increased with age until approximately 70 years, and females exhibited a significantly higher incidence than males in the over 50s age group (Figure 4).

Pearson’s correlation analysis was used to analyze the correlation between population characteristics and the incidence, death, reinfection, and other febrile diseases infection. The analysis revealed that increases in incidence were significantly correlated with higher population density, number of farmers, number of people aged over 60, and average age. However, no significant correlation was found with the average age of scrub typhus cases. Similarly, the number of individuals with reinfection who died was significantly correlated with population density, average age, the number of farmers and over 60, and additional infections of other febrile disease were significantly associated with all factors, including the average age of scrub typhus cases (Table 3).

In the Chungcheong region, a total of 123 cases were infected more than once from 2012 to 2022, with 85 in Chungnam, 25 in Daejeon, 3 in Sejong, and 10 in the Chungbuk area.

### 3.5. Analyze Risk Factors of Scrub Typhus Case

Logistic regression analysis was conducted to identify the risk factors for scrub typhus. The relative risks in the Sejong and Chungnam areas were 1.3 and 1.7 times higher than in the Daejeon area, respectively, while the risk in the Chungbuk area was lower. When analyzed by sex, the relative risk was 1.3 times higher in females than in males. Regarding the age groups, individuals aged over 19 years had a significantly higher risk compared with those aged under 12 years group, while those aged over 75 years had a 44 times higher risk (Table 4). No significant differences were found in the 13–19 years age group. In 2013, the relative risk significantly increased by 1.1 times compared with that in 2012, and after 2014, the risk decreased over the years. The relative risk increased slightly in 2021–2022 compared to 2020, but it was still lower than in 2012 (Table 5). 

**Figure 5:**
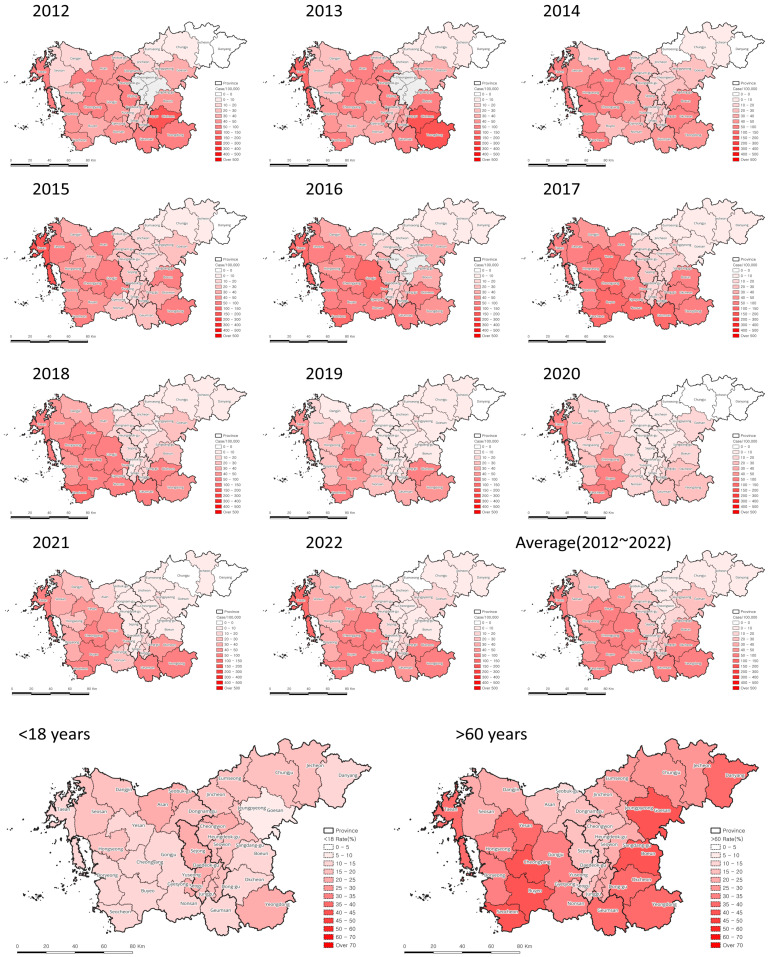
Geographical distribution of incidence (per 100,000 people) of scrub typhus (2012–2022).

When identifying the geographical distribution of scrub typhus cases by year in the Chungcheong region, the incidence rate was consistently high in south and western areas from 2012 to 2022. In the Sejong area, a high incidence rate was reported from 2012 to 2013, which subsequently decreased. The high-incidence area was mostly consistent with regions with a high proportion of over 60s. This suggests that a high proportion area of older people is high-risk area of scrub typhus (Figure 5).

## 4. Discussion

Scrub typhus, a significant febrile disease prevalent during the fall season, demonstrated a steady increase from 2012 to 2016, reaching 10,000 cases in 2016 [1]. Subsequently, the constructive prevention and promotion policies contributed to a decline in the number of cases, but a slight increase has been observed after 2020. The Chungcheong region, particularly the Chungnam area, exhibits a higher incidence rate compared with other regions, which requires intensive analysis (Figure 4). However, existing research on scrub typhus in the Chungcheong region has predominantly investigated the clinical characteristics of pediatric cases [21,22].

The Sejong area had a high incidence rate from 2012 to 2013, but it significantly decreased since 2014. Conversely, the Chungnam area continues to experience a high incidence rate. The construction of the administrative center complex in the Sejong area has attracted a large number of young peoples, resulting in a decreased agricultural population and a clear distinction between urban and suburban areas [23]. The average age dropped from 40.1 years in 2012 to 38.1 years in 2022, even reaching 36.7 years in 2017 (Figure 2). This urbanization and reduction in the average age likely contributed to the decrease in the incidence of scrub typhus in the Sejong area. Specifically, the average age of cases with scrub typhus also decreased significantly from 2016 to 2017; afterwards, the average age rapidly decreased in 2014–2015 in Sejong [27]. Since then, the average age of people and cases have steadily increased (Figure 1). 

This trend indicates that the average case age is increasing, which could lead to increased mortality and severity [24,25]. This aging may correlate with a slight increase in deaths by 2022 (Table 2). Previous studies have shown that tsutsugamushi infection is associated with increasing rates of dementia and other complications [6], and older adults almost always have underlying diseases. Therefore, targeted measures are needed to reduce its severity.

The correlation between increasing age and the increasing number of deaths was statistically confirmed. Both age and average age were strongly associated with the number of deaths, scrub typhus cases, reinfections, and additional infection with other febrile disease (Table 3). 

The geographical distribution of scrub typhus cases in the Chungcheong region also showed a high incidence in areas with a high proportion of over 60s, suggesting that more attention should be paid to outbreak prevention in areas with a large number of older people (Figure 5). Additionally, this indicates that high incidence rates may occur in some northeastern areas, where the proportion of over 60s or older is high, but the current incidence rate is low [26]. 

The risk analysis of the Chungcheong region showed that the incidence of scrub typhus was higher in the Chungnam area, female, and over 75 years, and it was higher than that in high-risk age groups in Taiwan (50–59 years) and Vietnam (41–50 years) [21,22]. In 2020–2022, the risk by age group was significantly higher by 92 times in those over 75 years than in those 0–12 years group. A slight increase in relative risk was observed after 2021, although the risk still remained lower than that in 2012–2013 (Table 4 and Table 5).

In conclusion, the high-incidence period in the Chungcheong region was similar to the results in other countries. Increased risk has been identified during periods of high mite density or increased agricultural activity [20]. 

Incidence of scrub typhus has been decreasing since 2017, but the relative risk and incidence have increased over the last 2 years (2021–2022). The relative risk analysis correlation with the incidence and other factors in the Chungcheong region showed concentrations in the Chungnam area, among females, and for those over 75 years. In addition, the age group and the average age of scrub typhus cases was higher than those in other countries in Asia [21,22].

In particular, the Sejong area showed a significant decrease in the number of cases due to various changes in population characteristics, including a decrease in the average age and agricultural population due to the influx of younger generations following the construction of a multifunctional administrative city. It was statistically confirmed that changes in the average age, the proportion of elderly people, and the agricultural population were associated with the incidence of scrub typhus. The risk of additional tick-borne febrile diseases (SFTS, Lyme disease, leptospirosis, and murine typhus) were also significantly higher among older people and agricultural populations, which is thought to be due to the higher probability of mite exposure in rural areas. These results were similar with other reports that agricultural population, forest density, and deforestation have affected the occurrence of scrub typhus cases in Korea [23]. 

In addition to these demographic changes, the decrease in scrub typhus incidence in 2017–2018 was also attributed to the implementation of aggressive public relations policies. These included prevention video clips for high-risk groups and distribution of protective gear, repellents, etc. Due to the subsequent COVID-19 pandemic, the number of people with scrub typhus has continued to decline. However, the number of cases is gradually increasing due to the increase in outdoor activity, and the possibility of severity is also increasing due to the aging of cases. In addition, cases of reinfection or additional infection with other febrile diseases cases also increase with age. 

Although the risk of occupational exposure is decreasing as the agricultural population declines, recent research trends show that outbreaks are increasingly caused by outdoor activities, such as leisure activities, camping, and weekend farms, rather than agricultural activities [11,28]; therefore, improved publicity strategies and policies are needed. 

Older people tend to live in rural areas with high mite densities and have less hygiene awareness than younger people. Female, including housewives, are more likely than male to be exposed to ticks through gardening and outdoor activities. Agricultural populations are traditionally the most likely occupational group to be exposed to mites. 

These high-risk groups need to be vigilant about exposure to mites during outdoor and agricultural activities, and should receive active health promotion to minimize their risk.

This study monitored long-term outbreaks in the Chungcheong region and identified the associations between scrub typhus and changes in demographic characteristics. However, further analysis using nationwide sample data and demographic characteristics is required. Nevertheless, this study provides basic information for the development of customized policies for preventing the occurrence of scrub typhus in the Chungcheong region. This includes: (1) Personalized health promotion based on exposure to agriculture and outdoor activities, (2) Focused prevention education for older adults, and (3) Regionalized health promotion based on local characteristics such as average age, percentage of agricultural land (Table S1) and other factors.

## 5. Conclusions

The incidence of scrub typhus in the Chungcheong region has been decreasing since 2017; however, it has been gradually increasing since 2021 with the aging of the population. Although the proportion of the agricultural population considered a high-risk group is decreasing, infections attributed to outdoor activities, such as camping and weekend farming, are on the rise. This alteration may lead to increased mortality and severity, indicating the need for changes in prevention promotion policies.

## Figures and Tables

**Table 1:** General characteristics of individuals with scrub typhus in Chungcheong region (2012–2022). Number of cases (%).

		2012	2013	2014	2015	2016	2017	2018	2019	2020	2021	2022
**Total**		1632	1993	1425	1412	1638	1853	1315	667	581	883	980
**Area**	Daejeon	444	517	300	284	307	297	182	117	113	160	175
		(27.2)	(25.9)	(21.1)	(20.1)	(18.7)	(16.0)	(13.8)	(17.5)	(19.4)	(18.1)	(17.9)
	Sejong	88	69	49	42	73	65	43	12	30	33	44
		(5.4)	(3.5)	(3.4)	(3.0)	(4.5)	(3.5)	(3.3)	(1.8)	(5.2)	(3.7)	(4.5)
	Chungnam	738	1,010	819	858	1,006	1,250	907	442	371	571	645
		(45.2)	(50.7)	(57.5)	(60.8)	(61.4)	(67.5)	(69.0)	(66.3)	(63.9)	(64.7)	(65.8)
	Chungbuk	362	397	257	228	252	241	183	96	67	119	116
		(22.2)	(19.9)	(18.0)	(16.1)	(15.4)	(13.0)	(13.9)	(14.4)	(11.5)	(13.5)	(11.8)
**Sex**	Male	606	748	552	520	693	754	486	260	217	323	410
		(37.1)	(37.5)	(38.7)	(36.8)	(42.3)	(40.7)	(37.0)	(39.0)	(37.3)	(36.6)	(41.8)
	Female	1,026	1,245	873	892	945	1,099	829	407	364	560	570
		(62.9)	(62.5)	(61.3)	(63.2)	(57.7)	(59.3)	(63.0)	(61.0)	(62.7)	(63.4)	(58.2)
**Age**	0–12	31	29	9	12	18	22	7	5	4	5	4
		(1.9)	(1.5)	(0.6)	(0.8)	(1.1)	(1.2)	(0.5)	(0.7)	(0.7)	(0.6)	(0.4)
	13–18	18	11	15	4	15	12	3	3	2	5	3
		(1.1)	(0.6)	(1.1)	(0.3)	(0.9)	(0.6)	(0.2)	(0.4)	(0.3)	(0.6)	(0.3)
	19–39	140	111	63	78	84	88	47	33	25	33	29
		(8.6)	(5.6)	(4.4)	(5.5)	(5.1)	(4.7)	(3.6)	(4.9)	(4.3)	(3.7)	(3.0)
	40–59	495	643	434	362	428	516	320	136	102	157	153
		(30.3)	(32.3)	(30.5)	(25.6)	(26.1)	(27.8)	(24.3)	(20.4)	(17.6)	(17.8)	(15.6)
	60–74	607	788	578	610	656	701	534	261	251	359	441
		(37.2)	(39.5)	(40.6)	(43.2)	(40.0)	(37.8)	(40.6)	(39.1)	(43.2)	(40.7)	(45.0)
	>75	341	411	326	346	437	514	404	229	197	324	350
		(20.9)	(20.6)	(22.9)	(24.5)	(26.7)	(27.7)	(30.7)	(34.3)	(33.9)	(36.7)	(35.7)
**Occupation**	Housewife	220	246	181	157	172	240	179	60	47	64	45
		(13.5)	(12.3)	(12.7)	(11.1)	(10.5)	(13.0)	(13.6)	(9.0)	(8.1)	(7.2)	(4.6)
	Soldier	10	17	5	4	9	7	3	2	0	1	0
		(0.6)	(0.9)	(0.4)	(0.3)	(0.5)	(0.4)	(0.2)	(0.3)	(0.0)	(0.1)	(0.0)
	Engineer	22	23	20	8	18	14	14	2	2	7	5
		(1.3)	(1.2)	(1.4)	(0.6)	(1.1)	(0.8)	(1.1)	(0.3)	(0.3)	(0.8)	(0.5)
	Farmer	245	350	220	250	298	359	222	111	60	64	79
		(15.0)	(17.6)	(15.4)	(17.7)	(18.2)	(19.4)	(16.9)	(16.6)	(10.3)	(7.2)	(8.1)
	Officer	66	44	38	23	18	19	19	6	4	6	4
		(4.0)	(2.2)	(2.7)	(1.6)	(1.1)	(1.0)	(1.4)	(0.9)	(0.7)	(0.7)	(0.4)
	Service	28	43	19	34	25	23	18	9	8	3	11
		(1.7)	(2.2)	(1.3)	(2.4)	(1.5)	(1.2)	(1.4)	(1.3)	(1.4)	(0.3)	(1.1)
	Sales	10	15	7	7	5	6	5	6	2	1	1
		(0.6)	(0.8)	(0.5)	(0.5)	(0.3)	(0.3)	(0.4)	(0.9)	(0.3)	(0.1)	(0.1)
	Student	32	29	22	16	21	25	6	9	7	8	2
		(2.0)	(1.5)	(1.5)	(1.1)	(1.3)	(1.3)	(0.5)	(1.3)	(1.2)	(0.9)	(0.2)
	Labor	15	19	18	16	17	18	9	7	3	0	2
		(0.9)	(1.0)	(1.3)	(1.1)	(1.0)	(1.0)	(0.7)	(1.0)	(0.5)	(0.0)	(0.2)
	Specialized job	3	4	8	5	1	4	3	3	2	1	1
		(0.2)	(0.2)	(0.6)	(0.4)	(0.1)	(0.2)	(0.2)	(0.4)	(0.3)	(0.1)	(0.1)
	Not employed	295	332	241	337	369	431	319	135	124	192	138
		(18.1)	(16.7)	(16.9)	(23.9)	(22.5)	(23.3)	(24.3)	(20.2)	(21.3)	(21.7)	(14.1)
	Etc. *	686	871	646	555	685	707	518	317	322	536	692
		(42.0)	(43.7)	(45.3)	(39.3)	(41.8)	(38.2)	(39.4)	(47.5)	(55.4)	(60.7)	(70.6)

* Includes fisher, restaurants, part-time jobs, freelancers, security guards, drivers’ personal businesses and other jobs.

**Table 2:** General characteristics of individuals who died of scrub typhus in Chungcheong region (2012–2022). (Number of patients).

		2012	2013	2014	2015	2016	2017	2018	2019	2020	2021	2022
**Region**	**Total**	2	5	0	0	1	4	1	0	1	1	7
	**Daejeon**	0	0	0	0	0	0	0	0	0	0	1
	**Sejong**	0	0	0	0	0	0	0	0	0	0	0
	**Chungnam**	2	3	0	0	1	2	1	0	1	1	5
	**Chungbuk**	0	2	0	0	0	2	0	0	0	0	1
**Sex**	**Male**	0	3	0	0	0	2	1	0	0	1	6
	**Female**	2	2	0	0	1	2	0	0	1	0	1
**Age**	**40–** **59**	0	1	0	0	0	0	1	0	0	0	0
	**60–** **74**	0	1	0	0	0	0	0	0	0	0	0
	**>75**	2	3	0	0	1	4	0	0	1	1	7
	**Mean**	79.5	69.4	-	-	88.0	81.0	47.0	-	79.0	78.0	82.4
**Cause of Death**	**Scrub typhus**	1	2	0	0	0	0	0	0	0	0	3
	**Sepsis**	0	2	0	0	0	0	0	0	1	0	3
	**Respiratory Failure**	0	0	0	0	0	1	0	0	0	0	1
	**Multiple Organ Failure**	1	1	0	0	1	3	1	0	0	1	0
**Day from Symptom to Death**		1–6	22 *	-	-	4	1–14	10	-	2	4	1–15

* Only partial cases (1 of 5 cases) included.

**Table 3:** Pearson correlation analysis of the association between incidence and demographical characteristics.

	Population	Number of Farmer	Over 60 Years	Average Age	Average Age of Case
**Scrub Typhus**	0.712 **	0.776 **	0.635 **	0.306 *	−0.105
**Reinfection**	0.476 **	0.39 3 *	0.553 **	0.447 **	0.141
Other Disease ***	0.477 **	0.470 **	0.638 **	0.601 **	0.396 **
**Death**	0.431 **	0.530 **	0.503 **	0.402 **	0.220

* 95% significant level (<0.05) ** 99% significant level (<0.01) *** Other disease: Severe febrile with thrombocytopenia syndrome (SFTS), Lyme disease, leptospirosis, Murine typhus.

**Table 4:** Logistic regression analysis of the relative risk of scrub typhus case by year.

Univariate	2012–2014	2015–2017	2018–2020	2021–2022
**Region**				
**Daejeon**	ref.	ref.	ref.	ref.
**Sejong**	1.682 ** (1.451–1.949)	1.408 ** (1.199–1.653)	1.139 (0.902–1.439)	1.171 (0.914–1.500)
**Chungnam**	1.254 ** (1.172–1.342)	2.119 ** (1.966–2.284)	2.458 ** (2.206–2.737)	2.087 ** (1.849–2.356)
**Chungbuk**	0.670 ** (0.671–0.728)	0.664 ** (0.602–0.733)	0.668 ** (0.579–0.771)	0.546 ** (0.462–0.645)
**Sex**				
**Male**	ref.	ref.	ref.	ref.
**Female**	1.443 ** (1.363–1.528)	1.295 ** (1.222–1.371)	1.424 ** (1.314–1.544)	1.310 * (1.193–1.439)
**Age**				
**0–12**	ref.	ref.	ref.	ref.
**13–18**	1.051 (0.720–1.535)	1.083 (0.694–1.690)	0.983 (0.421–2.296)	1.643 (0.634–4.258)
**19–39**	2.028 ** (1.563–2.632)	2.138 ** (1.586–2.882)	2.866 ** (1.694–4.851)	2.921 * (1.452–5.877)
**40–59**	9.224 ** (4.81–10.309)	9.708 ** (7.357–12.808)	12.703 ** (7.728–20.881)	11.552 ** (5.954–22.413)
**60–75**	29.947 ** (22.769–36.803)	33.244 ** (25.240–43.786)	47.060 ** (28.720–77.110)	51.661 ** (12.897–206.938)
**>75**	31.968 ** (25.055–40.788)	41.861 ** (31.718–55.246)	71.694 ** (43.704–117.610)	92.737 ** (48.327–179.067)

* 95% significant level (<0.05), ** 99% significant level (<0.01). ref, reference.

**Table 5:** Relative risk prediction through the logistic regression analysis of various factors.

	Univariate				Multivariate			
	β	RR *	(95% CI)	P	β	RR *	(95% CI)	P
**Region**								
**Daejeon**	ref.	1.000			ref.	1.000		
**Sejong**	0.074	1.077	0.113	0.983–1.180	0.287	1.332	<0.01	1.216–1.460
**Chungnam**	0.754	2.125	<0.01	2.037–2.216	0.579	1.785	<0.01	1.711–1.862
**Chungbuk**	−0.283	0.753	<0.01	0.713–0.796	−0.431	0.650	<0.01	0.616–0.687
**Sex**								
**Male**	ref.	1.000			ref.	1.000		
**Female**	0.479	1.614	<0.01	1.561–1.669	0.314	1.368	<0.01	1.323–1.415
**Age**								
**0** **–** **12**	ref.	1.000			ref.	1.000		
**13** **–** **18**	0.104	1.110	0.434	0.854–1.442	0.100	1.105	0.456	0.850–1.435
**19** **–** **39**	0.769	2.157	<0.01	1.806–2.576	0.795	2.214	<0.01	1.854–2.644
**40** **–** **59**	2.252	9.506	<0.01	8.058–11.216	2.291	9.885	<0.01	8.378–11.662
**60** **–** **75**	3.460	31.827	<0.01	27.006–37.508	3.523	33.811	<0.01	28.748–39.931
**>75**	3.822	45.693	<0.01	38.374–53.903	3.806	44.986	<0.01	38.128–53.077
**Year**								
**2012**	ref.	1.000			ref.	1.000		
**2013**	0.192	1.211	<0.01	1.135–1.293	0.171	1.187	<0.01	1.112–1.267
**2014**	–0.154	0.857	<0.01	0.798–0.920	–0.199	0.820	<0.01	0.764–0.880
**2015**	–0.175	0.840	<0.01	0.782–0.902	–0.244	0.784	<0.01	0.730–0.842
**2016**	–0.036	0.964	0.299	0.900–1.033	–0.129	0.879	<0.01	0.821–0.942
**2017**	–0.078	1.081	<0.01	1.012–1.156	–0.041	0.960	0.224	0.898–1.026
**2018**	–0.271	0.762	<0.01	0.709–0.820	–0.415	0.660	<0.01	0.614–0.710
**2019**	–0.952	0.386	<0.01	0.353–0.422	–1.125	0.325	<0.01	0.297–0.355
**2020**	–1.090	0.336	<0.01	0.306–0.369	–1.295	0.274	<0.01	0.249–0.301
**2021**	–0.672	0.511	<0.01	0.471–0.554	–0.905	0.405	<0.01	0.373–0.439
**2022**	–0.569	0.566	<0.01	0.523–0.613	–0.828	0.437	<0.01	0.404–0.473

* RR, risk ratio; ref, reference.

## Data Availability

All data related to the research are included in the article.

## Supplementary Materials

The following supporting information can be downloaded at: https://www.mdpi.com/article/10.3390/tropicalmed9070147/s1, Figure S1: General information of the Chungcheong region; Figure S2: Population changes following region and years; Table S1: Change in the land of agricultural land (2012–2022) (ha: 10,000 m^2^).
