# Supplementary material for: Incidence of Scrub Typhus according to Changes in Geographic and Demographic Characteristic in the Chungcheong Region of Korea
Source: Trop Med Infect Dis. 2024 Jun 30;9(7):147. doi: 10.3390/tropicalmed9070147 (PMC11281401; doi:10.3390/tropicalmed9070147)
Supplement: Supplementary file 1 [file tropicalmed-09-00147-s001.zip › tropicalmed-3056935-supplementary.pptx]

## Slide 1
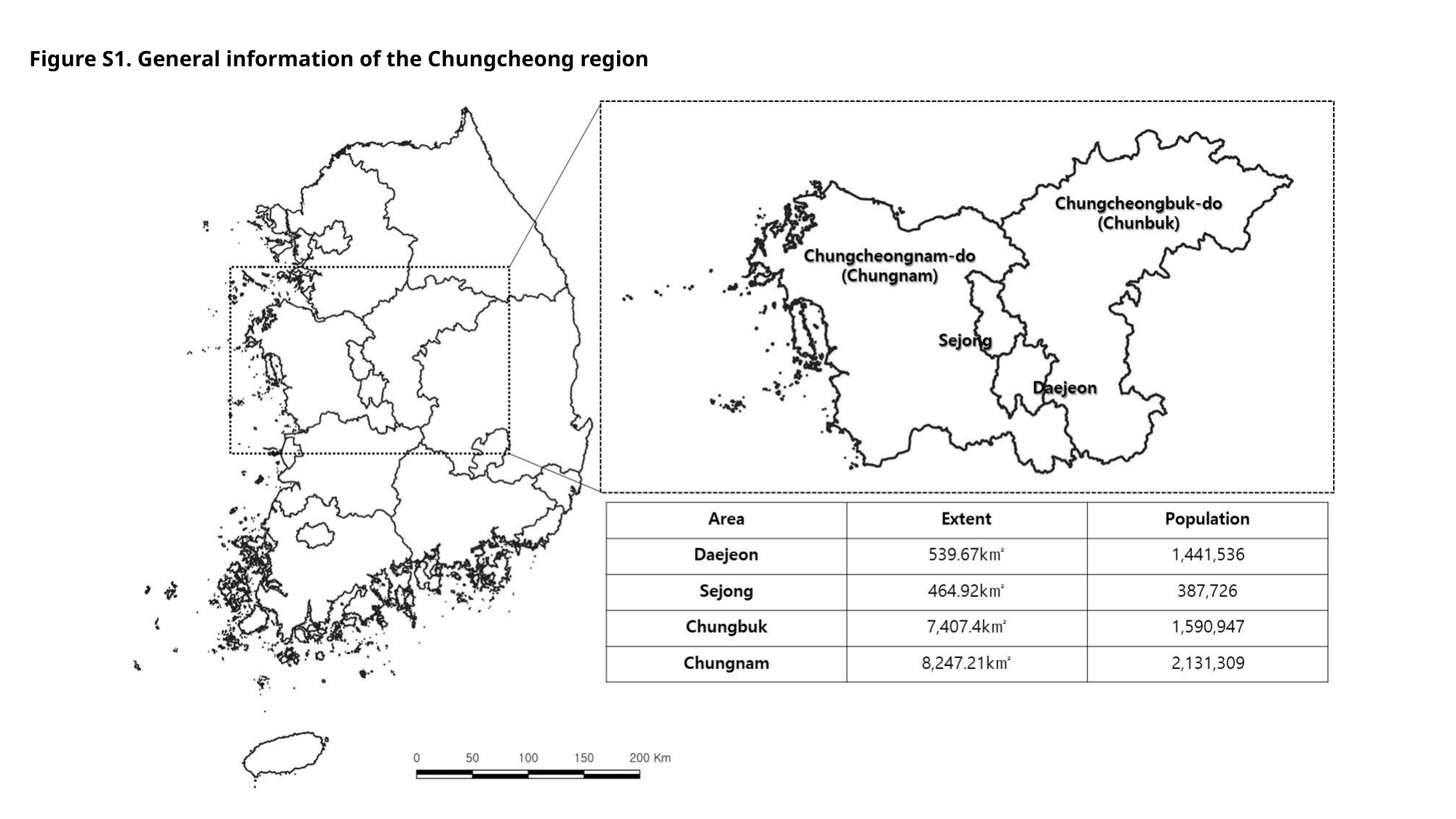

Figure S1. General information of the Chungcheong region
#

## Slide 2
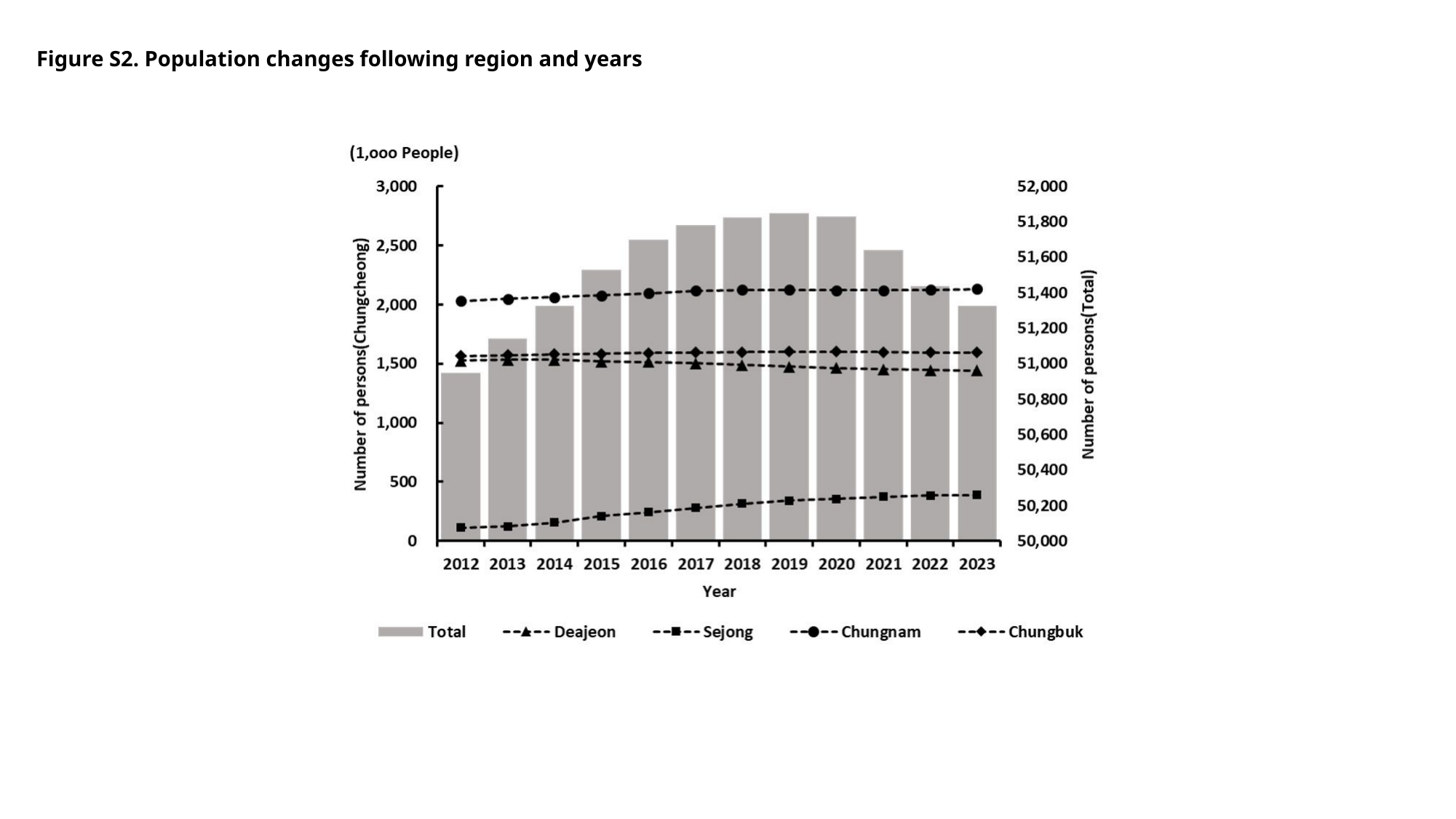

Figure S2. Population changes following region and years

## Slide 3
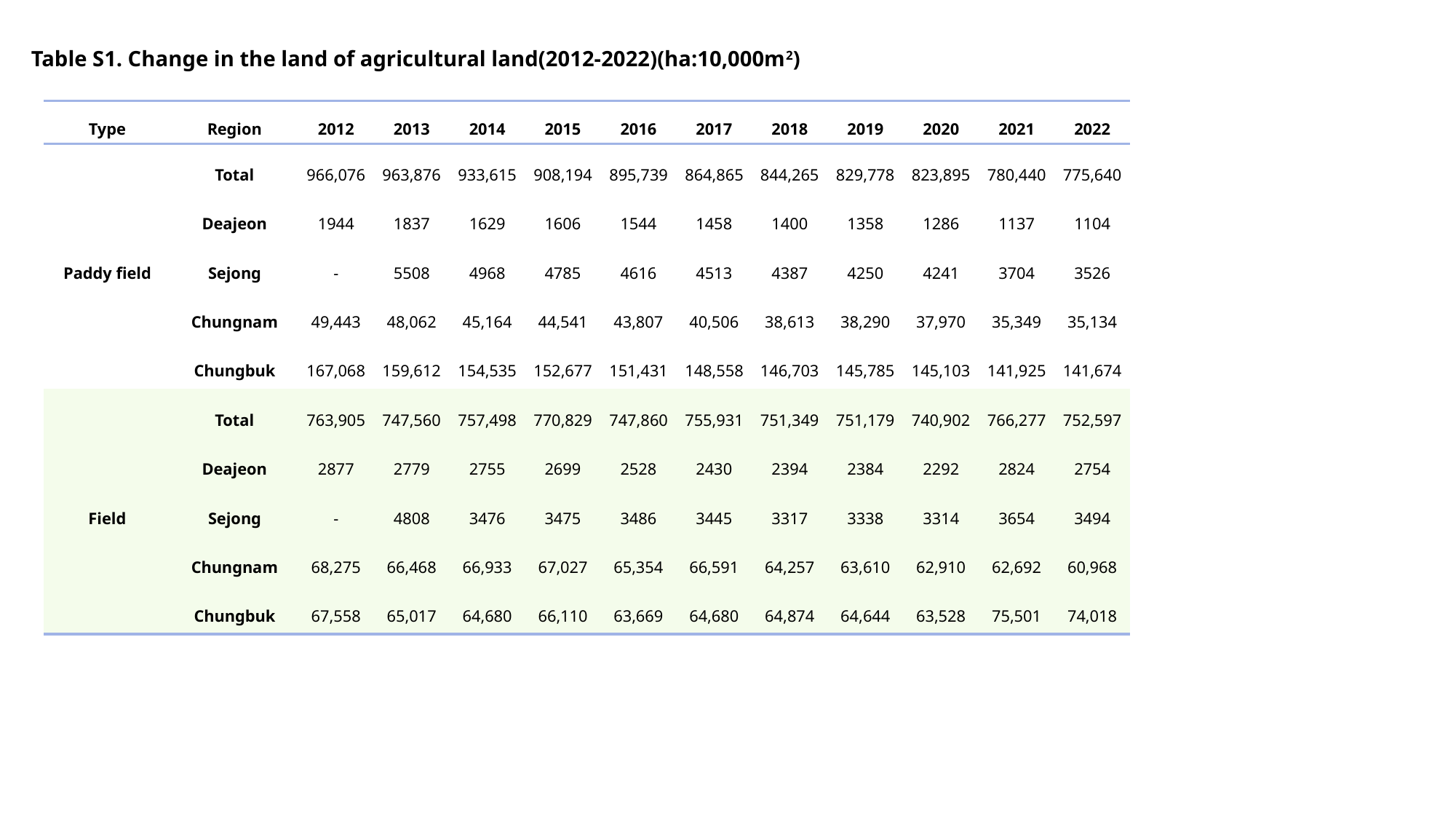

Table S1. Change in the land of agricultural land(2012-2022)(ha:10,000m2)
| Type | Region | 2012 | 2013 | 2014 | 2015 | 2016 | 2017 | 2018 | 2019 | 2020 | 2021 | 2022 |
| --- | --- | --- | --- | --- | --- | --- | --- | --- | --- | --- | --- | --- |
| Paddy field | Total | 966,076 | 963,876 | 933,615 | 908,194 | 895,739 | 864,865 | 844,265 | 829,778 | 823,895 | 780,440 | 775,640 |
| | Deajeon | 1944 | 1837 | 1629 | 1606 | 1544 | 1458 | 1400 | 1358 | 1286 | 1137 | 1104 |
| | Sejong | - | 5508 | 4968 | 4785 | 4616 | 4513 | 4387 | 4250 | 4241 | 3704 | 3526 |
| | Chungnam | 49,443 | 48,062 | 45,164 | 44,541 | 43,807 | 40,506 | 38,613 | 38,290 | 37,970 | 35,349 | 35,134 |
| | Chungbuk | 167,068 | 159,612 | 154,535 | 152,677 | 151,431 | 148,558 | 146,703 | 145,785 | 145,103 | 141,925 | 141,674 |
| Field | Total | 763,905 | 747,560 | 757,498 | 770,829 | 747,860 | 755,931 | 751,349 | 751,179 | 740,902 | 766,277 | 752,597 |
| | Deajeon | 2877 | 2779 | 2755 | 2699 | 2528 | 2430 | 2394 | 2384 | 2292 | 2824 | 2754 |
| | Sejong | - | 4808 | 3476 | 3475 | 3486 | 3445 | 3317 | 3338 | 3314 | 3654 | 3494 |
| | Chungnam | 68,275 | 66,468 | 66,933 | 67,027 | 65,354 | 66,591 | 64,257 | 63,610 | 62,910 | 62,692 | 60,968 |
| | Chungbuk | 67,558 | 65,017 | 64,680 | 66,110 | 63,669 | 64,680 | 64,874 | 64,644 | 63,528 | 75,501 | 74,018 |
